# Osteoporosis in Patients with Chronic Kidney Diseases: A Systemic Review

**DOI:** 10.3390/ijms21186846

**Published:** 2020-09-18

**Authors:** Chia-Yu Hsu, Li-Ru Chen, Kuo-Hu Chen

**Affiliations:** 1 Department of Rehabilitation Medicine, Ten-Chan General Hospital, Zhongli, Taoyuan 320, Taiwan; f927kimo@gmail.com; 2 Department of Biomedical Engineering, Chung Yuan Christian University, Taoyuan 320, Taiwan; 3 Department of Physical Medicine and Rehabilitation, Mackay Memorial Hospital, Taipei 104, Taiwan; gracealex168@gmail.com; 4 Department of Mechanical Engineering, National Chiao-Tung University, Hsinchu 300, Taiwan; 5 Department of Obstetrics and Gynecology, Taipei Tzu-Chi Hospital, The Buddhist Tzu-Chi Medical Foundation, Taipei 231, Taiwan; 6 Department of Medicine, School of Medicine, Tzu-Chi University, Hualien 970, Taiwan

**Keywords:** chronic kidney disease, osteoporosis, dialysis, fracture

## Abstract

Chronic kidney disease (CKD) is associated with the development of mineral bone disorder (MBD), osteoporosis, and fragility fractures. Among CKD patients, adynamic bone disease or low bone turnover is the most common type of renal osteodystrophy. The consequences of CKD-MBD include increased fracture risk, greater morbidity, and mortality. Thus, the goal is to prevent the occurrences of fractures by means of alleviating CKD-induced MBD and treating subsequent osteoporosis. Changes in mineral and humoral metabolism as well as bone structure develop early in the course of CKD. CKD-MBD includes abnormalities of calcium, phosphorus, PTH, and/or vitamin D; abnormalities in bone turnover, mineralization, volume, linear growth, or strength; and/or vascular or other soft tissue calcification. In patients with CKD-MBD, using either DXA or FRAX to screen fracture risk should be considered. Biomarkers such as bALP and iPTH may assist to assess bone turnover. Before initiating an antiresorptive or anabolic agent to treat osteoporosis in CKD patients, lifestyle modifications, such as exercise, calcium, and vitamin D supplementation, smoking cessation, and avoidance of excessive alcohol intake are important. Managing hyperphosphatemia and SHPT are also crucial. Understanding the complex pathogenesis of CKD-MBD is crucial in improving one’s short- and long-term outcomes. Treatment strategies for CKD-associated osteoporosis should be patient-centered to determine the type of renal osteodystrophy. This review focuses on the mechanism, evaluation and management of patients with CKD-MBD. However, further studies are needed to explore more details regarding the underlying pathophysiology and to assess the safety and efficacy of agents for treating CKD-MBD.

## 1. Overview

Chronic kidney disease (CKD) is associated with the development of mineral bone disorder (MBD), osteoporosis, and fragility fractures. The grade or severity of CKD can be classified based on glomerular filtration rate (GFR) category, ranging from stage G1 to G5 [1]. In stage G1 and G2, the GFRs are normal (more than 90 mL/min/1.73 m^2^) and slightly decreased (60–89 mL/min/1.73 m^2^), respectively. For patients with CKD stage G3a and G3b, the GFRs decrease from mild to moderate (40–59 mL/min/1.73 m^2^) and moderate to severe (30–44 mL/min/1.73 m^2^). The renal function is severely impaired in patients with CKD stage 4 (15–29 mL/min/1.73 m^2^), and CKD stage 5 (add D if treated by dialysis) referring to kidney failure (less than 15 mL/min/1.73 m^2^) [1]. By definition, CKD-MBD is a systemic disorder of mineral and bone metabolism, which is manifested by either one or a combination of the following: abnormalities of calcium, phosphorus, parathyroid hormone (PTH), or vitamin D metabolism; abnormalities in bone turnover, mineralization, volume, linear growth, or strength; vascular or other soft tissue calcification [2]. The consequences of CKD-MBD include increased fracture risk, greater morbidity, and mortality [3].

Osteoporosis is a skeletal disorder characterized by compromised bone strength predisposing to an increased risk of fracture [4]. The World Health Organization (WHO) defines osteoporosis as a T score ≤ −2.5. CKD is an independent risk factor of osteoporosis [5]. The prevalence of osteoporosis was 31.8% among CKD G3–5 patients in the Kashmir valley [5]. Among the National Health and Nutrition Examination Survey (NHANES Ⅲ) participants, osteoporosis was twice as common in those with eGFR < 60 mL/min than those with eGFR > 60 mL/min [6]. As CKD makes progress, decreased bone mineral density (BMD) mostly involves hip, but not spine [7].

CKD is a risk factor for fragility (low trauma) fractures [8,9]. The Longitudinal Aging Study Amsterdam (LASA) survey found that early decreased renal function (eGFR< 60 mL/min/1.73 m^2^) was related to increase incident fracture risk, but not with increased prevalence of vertebral fracture or falls [10]. In a prospective cohort study in veterans with CKD G3–5, CKD is related to a moderately greater fracture risk after adjusting age, race, and BMD [11]. In patients with CKD G5D and kidney transplant (KT), the incidence of hip fracture in hemodialysis (HD) group was higher than that in peritoneal dialysis (PD) or KT groups [3]. The consequence of fractures increased the mortality rate in CKD patients with non-dialysis [9] and with dialysis [12]. Thus, the goal is to prevent the occurrence of fractures by means of alleviating CKD-induced MBD and treating subsequent osteoporosis. This review focuses on the evaluation and management of patients with CKD-MBD.

## 2. Materials and Methods

This review was modeled based on the Preferred Reporting Items for Systematic Reviews and Meta-Analyses (PRISMA) statement. All of the articles were retrieved from the databases Medline and PubMed using the search terms “chronic kidney disease”, “osteoporosis”, “dialysis” and “fracture” for the topic. For further screening and selection, only full-text articles were considered to be included for a further analysis. A literature search was conducted on Medline and PubMed databases up to 30 June 2020 to identify all potential articles in the data sources. In the screening process, duplication articles and research before 1980 were excluded. After initial processing, two experts in the field independently reviewed potentially eligible studies for exclusion and inclusion. Articles with poor study design or not-matched outcomes were deemed not eligible for the study. During the period of study selection, disagreements between the two reviewers were resolved by mutual discussion until a consensus was reached.

## 3. Search Results

**Figure 1:**
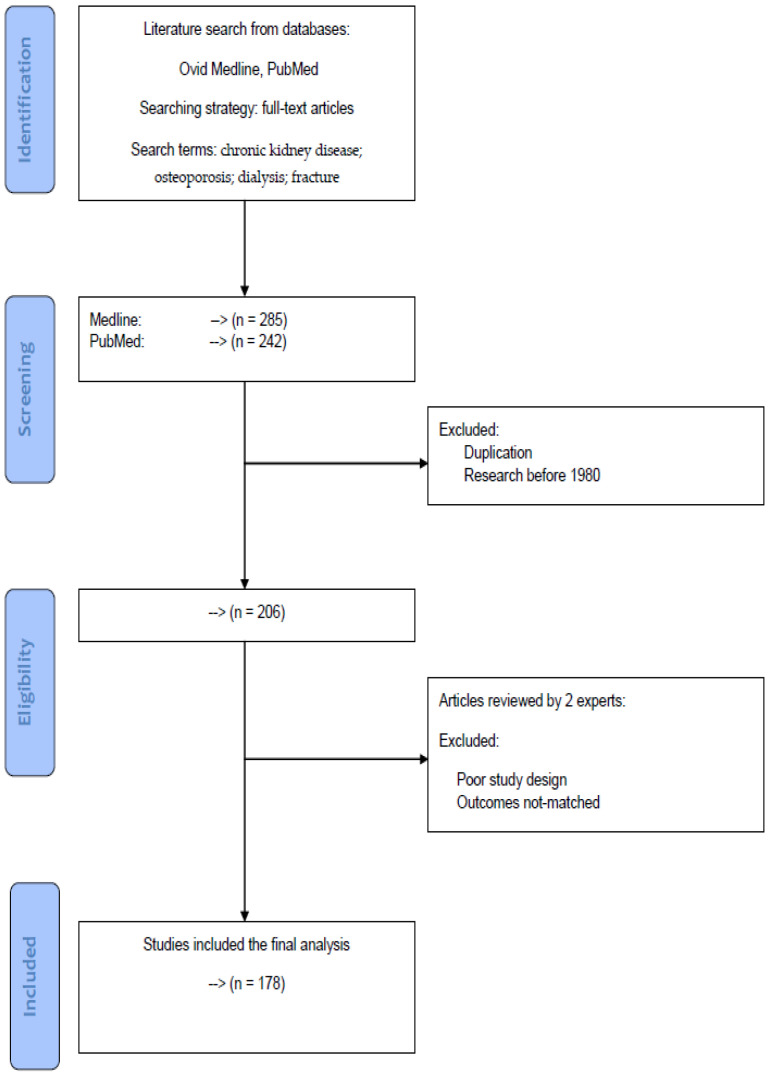
A flowchart of article selection to illustrate the processes of database identification, article screening, consideration of eligibility and final inclusion according to the PRISMA statement.

A flowchart of article selection has been shown in Figure 1 to illustrate the processes of database identification, article screening, consideration of eligibility and final inclusion according to the PRISMA statement. Using the search terms and strategy mentioned above, there were 285 (Medline) and 242 (PubMed) articles in the topic. After the screening process to exclude duplication articles and research before 1980, 206 articles were considered for further analysis. Each article was evaluated and extracted independently by two reviewers to inspect the sources including year, authors, research design and clinical outcomes. Differences in selection between the two reviewers were reassessed and discussed until a consensus was reached. After excluding articles with poor study design or not-matched outcomes, there were 178 eligible articles for the topic.

## 4. Characteristics of Chronic Kidney Disease-Mineral and Bone Disorder (CKD-MBD)

CKD-MBD describes abnormalities in mineral metabolism, skeletal health, and soft tissue calcifications. Possible factors involved in CKD-associated osteoporosis are summarized in Table 1. Kloth is a membrane protein expressed mainly in proximal and distal renal tubules and could be detected in blood, urine, cerebrospinal fluid and osteocytes. In early stage of CKD, the decline of Kloth expression can increase FGF-23 levels [13]. The effects of increase in FGF-23 levels lead to increased urinary phosphate excretion by reducing renal phosphate reabsorption [14] and decreased calcitriol synthesis by inhibiting the proximal tubular expression of 1-alpha-hydroxylase enzyme [14,15]. Furthermore, sclerostin (28 kD) and dickkopf1 (26 kD) and are small secreted glycoproteins. As CKD progress, the consequences of increased serum sclerostin lead to deceased bone formation by inhibiting Wnt-induced signaling through binding to LRP5/6 [14,16] and increased osteoclastogenesis by inducing RANK-L synthesis [17]. The elevated dickkopf1 level could also inhibit bone formation by inhibiting Wnt-induced signaling through binding to LRP5/6 [16,18]. In early CKD, a tendency to phosphate retention plays a central role in the development of SPTH [19] and inhibits calcitriol synthesis by inhibiting the activity of 1-alpha-hydroxylase enzyme in kidney [20]. Moreover, the accumulation of uremic toxins, such as indoxyl sulfate and p-cresyl sulfate, could reduce the expression of PTH receptor in osteoblasts and is associated with skeletal resistance to PTH [21]. The fall of calcitriol concentrations could cause the increase of PTH secretion [20,22] and the decrease of serum calcium level [23]. Decreased free ionized calcium concentration could also accentuate SPTH [23]. Skeletal resistance to the calcemic effect on PTH may be involved in the development of SPTH in CKD [24]. Finally, SPTH is the major feature of CKD-MBD which causes abnormal bone remodeling and osteoporosis [23].

### 4.1. Abnormalities of Calcium, Phosphorus, PTH, or Vitamin D Metabolism

#### 4.1.1. Disorders of Calcium Balance

Hypocalcemia is common in CKD patients and contributes to increased PTH secretion and abnormal bone remodeling. The total serum calcium concentration decreases following phosphate retention, decreased 1,25(OH)_2_D (calcitriol) concentration, and resistance to the calcemic actions of PTH on bone during the process of CKD [23]. Serum calcium typically remain normal until eGFR decreased to 20 mL/min/1.73 m^2^ [25]. The decrease in serum calcium concentration is sensed by a specific membrane calcium-sensing receptor (CaSR) on parathyroid glands and is a potent stimulus for PTH release [26]. The decreased number and expression of CaSR in hypertrophied parathyroid glands may be related to the proliferation of parathyroid tissue [27], resulting in inadequate suppression of PTH by calcium and high PTH even in the setting of hypercalcemia. Thus, CaSR has a direct therapeutic implication in regulating parathyroid gland function by calcimimetics.

In patients with CKD G5D, both hypocalcemia and hypercalcemia are associated with mortality [28,29]. In patients after KT, hypercalcemia is common due to persistence of preexistent hyperparathyroidism, hypophosphatemia, postintervention immobilization, or progressive normalization of calcitriol level [30].

Calcium balance is whole-body calcium retention or deficit total calcium inputs subtract total body loses [31]. A positive balance may increase vascular calcification and cardiovascular events, while a negative balance may increase the risk of osteoporosis and fracture [31]. Serum calcium alone cannot serve as a proxy measurement of the whole-body calcium balance.

#### 4.1.2. Disorder of Phosphorus Metabolism

Phosphate retention begins early in CKD and plays a central role in the development of secondary hyperparathyroidism (SPTH) by inducing hypocalcemia, decreasing calcitriol synthesis, and increasing PTH gene expression [32,33]. However, serum phosphate levels are not usually elevated in the early stages of CKD because of a reduction in renal proximal tubular phosphate resorption owing to increased levels of PTH and fibroblast-growth-factor (FGF)-23 [15]. The effects of FGF-23 on increasing phosphate secretion may be blunted by decreased Kloth in early CKD. Serum phosphate typically remain normal until eGFR decreased to 20 mL/min/1.73 m^2^ [15,25]. In advanced CKD, the consequences of hyperphosphatemia included the stimulating effects on PTH synthesis and secretion [19], FGF-23 secretion [34], and osteoblastic transformation of the vascular smooth muscle cell which directly contributes to cardiovascular calcification [35]. In patients with CKD G5D, both low and high serum phosphate are associated with mortality [29].

#### 4.1.3. Disorder of Parathyroid Hormone Metabolism

Serum intact PTH (iPTH) typically remains normal until the eGFR decreases to approximately 45 mL/min/1.73 m^2^ [25,36]. The prevalence of SPTH increases as CKD progress [36]. The causes of increase in initiating and maintaining PTH included phosphate retention, decreased free ionized calcium level, decreased calcitriol level, increased FGF-23 level, and reduced expression of vitamin D receptors (VDRs), calcium-sensing receptors (CaSRs), FGF receptors and kloth in the parathyroid glands. Furthermore, uremic toxins such as indoxyl sulfate and p-presyl sulfate would reduce the expression of PTH receptor and production of PTH-induced cyclic adenosine 3′,5′ monophosphate in osteoblast, which lead to skeletal resistance to PTH and bone fragility [21]. In patients with CKD G5D, both low and high iPTH [28] are associated with mortality [29].

#### 4.1.4. Disorder of Vitamin D Metabolism

A serum 25(OH)D (calcidiol) level < 30 nmol/L indicates vitamin D deficiency [37,38], which is common among patients with CKD. Vitamin D deficiency is associated with cardiovascular events in CKD. One cross-sectional study reported that a high prevalence of calcidiol deficiency and insufficiency in patients with CKD G3-4 not on dialysis therapy [39]. Calcitriol level started to fall until eGFR was <40 mL/min/1.73 m^2^ [25]. In the course of eGFR decline, reductions in calcitriol levels occurred earlier than elevations in iPTH levels [25]. The primary cause why calcitriol level declines is the increase in FGF-23 concentration, rather than the loss of functioning renal tissue [15]. In patients with CKD-MBD undergoing HD, low serum calcidiol level was associated with bone-biopsy-proven increase of bone turnover [40].

### 4.2. Abnormalities in Bone Turnover, Mineralization, Volume, Volume Linear Growth, or Strength

#### 4.2.1. TMV Characteristics of Renal Osteodystrophy

Bone biopsy is the gold standard for the diagnosis and classification of bone diseases in CKD, or renal osteodystrophy. By definition, renal osteodystrophy is a noticeable transformation of bone morphology in patients with CKD; it is one measure of the skeletal component of the systemic disorder of CKD-MBD that is quantified by histomorphometry of bone biopsy [2]. Kidney Disease: Improving Global Outcomes (KDIGO) group recommends three parameters (bone turnover, mineralization, and volume; TMV system) to be used to assess bone pathology [1]. Bone turnover refers to the rate of skeletal remodeling, or the ratio between bone formation, and bone resorption [2]. Mineralization reflects how well bone collagen becomes calcified during the formation phase of skeletal remodeling [2]. Bone resorption is the main function of osteoclasts, which colonized fetal ossification centers coming from embryonic erythron-myeloid progenitors [41]. Volume indicates the amount of bone per unit volume of tissue [2]. Bone strengths is determined by bone quantity, and bone quality. Bone quality refers to the structure and material parameters that enables bone to bear load and resist fracture [42]. In TMV system used for evaluation of CKD-MBD, the bone turnover rates are high in patients with osteitis fibrosa cystica and mixed uremic osteodystrophy but low in patients with adynamic bone disease and osteomalacia [2]. The mineralization of bone is normal in patients with osteitis fibrosa cystica and adynamic bone disease but abnormal in patients with osteomalacia and mixed uremic osteodystrophy [2].

#### 4.2.2. Bone Turnover in CKD

##### Prevalence of Low-Turnover Bone Disease in CKD

The majority of renal osteodystrophy among CKD G5 and CKD G5D patients is a low-turnover bone disease (adynamic bone disease), particularly in diabetic patients [43,44,45]. Two large bone biopsy studies showed a high prevalence (58% and 59%) of adynamic bone disease in HD patients [46,47]. Prevalence of high-turnover bone disease (osteitis fibrosa cystica) among dialysis patients has markedly decreased. The incidence of osteomalacia has also been decreased with abandonment of aluminum-based phosphate binders and induction of efficient water treatment for preparing the dialysate [48]. Increased prevalence of low-turnover bone disease may be due to older age, increased number of diabetes patients, early use of vitamin D analogs and calcium-containing phosphate binders, and differences in dialysis techniques [48].

##### Evaluation of Bone Turnover

PTH levels are used as a surrogate to evaluate the status of bone turnover. Very high PTH levels (≥585 pg/mL) are usually associated with osteitis fibrosa [49], while very low PTH levels (<100 pg/mL) are associated with adynamic bone disease. A cross-sectional histomorphometric study of bone turnover showed that a PTH of >323 pg/mL had the best discriminatory ability of high from non-high bone turnover; a PTH < 103.8 pg/mL best discriminated low from non-low turnover [46]. However, PTH levels are not predictive of underlying bone disease when they are modestly elevated [50]. KDIGO suggests using PTH trends instead of absolute targets to guide treatment decision [1].

#### 4.2.3. Evaluation of Bone Strength

Dual-energy X-ray absorptiometry (DXA) evaluates the bone quantity and not the bone quality. Peripheral quantitative computed tomography (pQCT), high resolution pQCT (HRpQCT), and micromagnetic resonance imaging (microMRI) are non-invasive 3D imaging techniques that can detect microarchitecture and mineral density of both trabecular and cortical bones [51]. However, there are few data in evaluating these techniques in patients with CKD.

### 4.3. Vascular or Other Soft Tissue Calcification

Vascular calcification, which significantly increases cardiovascular and all-causes of mortality, is highly prevalent in hemodialysis patients. Extraskeletal calcification is common in patients with CKD G5D [52]. All arteries and arterioles can calcify, whereas veins hardly calcify unless injured or arterialized [53]. Among CKD patients, there are two types of vascular calcification: medial calcification and intimal calcification. Medial calcification is the result of both a phenotype switch of vascular smooth cells to osteoblast-like cells and local inflammation [54]. Intimal calcification is secondary to establish atherosclerosis. There are limited clinical data concerning the results of intimal versus medial lesions among CKD patients. One study of HD patients showed that patients with arterial media calcification had a longer survival than those with arterial intima calcification [55].

#### 4.3.1. Risk Factors

Risk factors for vascular calcification among CKD patients included increasing age [56,57,58], time on dialysis [58], hyperphosphatemia [57], persistent hypercalcemia, increased oral calcium intake [56,58], calcium-containing phosphate binders [58], secondary hyperparathyroidism [57], adynamic bone disease, untreated vitamin D deficiency [57], high dialysate calcium [59], hypomagnesemia [60], diabetes [61], dyslipidemia [58], and warfarin [62].

#### 4.3.2. Protectors 

Protectors for vascular calcification among CKD patients included Lanthanum carbonate [63], calcimimetic agent [64], kloth [13], pyrophosphate [65], osteoprotegerin [66], and RANK-L [66].

### 4.4. Evaluating Fracture Risk in CKD

Several longitudinal studies confirmed that low BMD could predict fractures in patients with CKD [67,68,69,70]. In patients with CKD G3a–G5D with evidence of CKD-MBD and/or risk factors for osteoporosis, KDIGO in 2017 suggest BMD testing to assess fracture risk if results will impact treatment decisions [1]. Although DXA is commonly used technique for quantifying BMD in CKD patients, it has some limitations. First, DXA measures areal BMD, not volumetric BMD. Second, it cannot discriminate between cortical and cancellous bone. Third, it cannot assess bone microarchitecture or bone turnover.

Fracture Risk Assessment Tool (FRAX) developed by WHO can also be helpful for predicting fracture risk in patients with non-dialysis CKD [71] and KT recipients [72]. However, the limitation of FRAX is that it does not include any adjustment of risk according to GFR.

**Figure 2:**
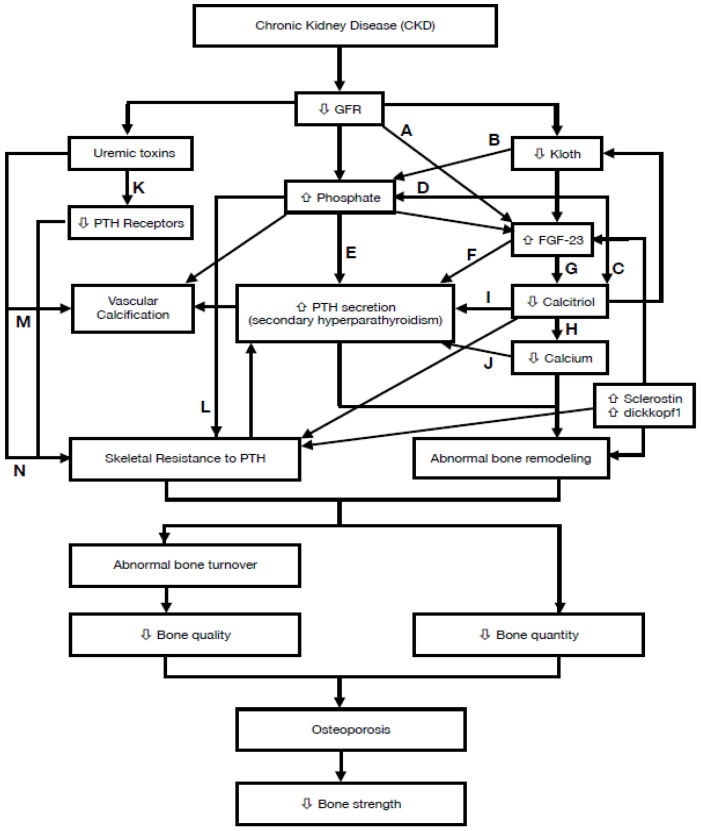
A summary of the mechanism underlying the effects of chronic kidney disease (CKD) on osteoporosis and subsequent decrease in bone strength. (A) decrease clearance of FGF-23 [15,73,74]; (B) decrease phosphaturic effect of FGF-23 [75]; (C) inhibit 1α-hydroxylase in proximal tubule of kidney [20]; (D) increase renal tubular resorption of phosphate [76]; (E) induce hypocalcemia, decrease calcitriol, increase PTH gene expression and PTH secretion [20,33,77]; (F) decrease its inhibitory effect of due to decreased FGFR1 and kloth protein [78,79]; (G) inhibit 1α-hydroxylase [15]; (H) decrease intestinal absorption of Ca and decrease Ca release from bone [22,80]; (I) decrease number of VDRs in parathyroid cells [81] and decrease its inhibitory effect on parathyroid gland [20,82]; (J) increase PTH mRNA concentration [19,83] and decreased expression of the calcium-sensing receptor (CaSR) on parathyroid gland [27,84,85]; (K) Indoxyl sulfate decreases the expression of PTH1 receptor [21]; (L) the physicochemical precipitation of inorganic phosphate and calcium can indirectly give rise to skeletal resistance to PTH; (M) Indoxyl sulfate increases CpG hypermethylation of the Kloth gene [86]; (N) P-cresyl sulfate induces osteoblastic dysfunction [87].

A summary of the mechanism underlying the effects of chronic kidney disease (CKD) on osteoporosis and subsequent decrease in bone strength is displayed in Figure 2. The reduction of GFR in patients with CKD initiates a cascade of metabolic disorder, abnormal bone remodeling and turnover, resulting in osteoporosis and subsequent decrease in bone strength.

## 5. Management of Chronic Kidney Disease-Mineral and Bone Disorder (CKD-MBD)

### 5.1. Lifestyle Modification

Non-pharmacologic interventions include modifying dietary calcium and nutritional vitamin D, increased physical activity, smoking cessation, weight bearing exercise, fall prevention [88], and avoidance of excessive alcohol intake [89,90].

### 5.2. Exercise and Physical Therapy

Exercise improves muscle impairment, physical function, and physical performance across the spectrum of CKD [91]. In a study of partial nephrectomy induced CKD rat model, exercise improved BMD and microstructure by inhibiting serum sclerostin level, therefore mitigating its effect of inhibiting Wnt/ß-catenin signaling pathway [92]. Exercise training prescriptions should be individualized to one’s physical function. According to FITT (frequency, intensity, time, and type) principle for exercise prescription, patients should exercise 2 to 3 times per week at the beginning of the training, and then increase frequency to 3 to 5 times per week. Intensity should be tailored based on patient tolerance to exercise. The duration of exercise relies on the health and physical condition of the patient. Type of exercise includes aerobic, resistance, and flexibility exercise [93].

### 5.3. Correction of Biochemical Abnormalities of CKD-MBD

#### 5.3.1. Phosphate

In patients with CKD G3a–G5D, the KDIGO group suggests lowering of hyperphosphatemia levels towards normal range [1]. Phosphate load from phosphate-rich sources should be avoided. Non-calcium-based phosphate binders, such as sevelamer, have advantages over calcium-based binder in increasing the bone formation rate and improving trabecular architecture [43].

#### 5.3.2. Calcium or Cinacalcet

Excessive exogenous calcium in adults may be harmful at all stages of CKD [31,94]. Updated 2017 KDIGO guidelines for CKD-MBD suggest limiting calcium-based phosphate binders for all patients with CKD G3a–5D [1]. Daily dietary calcium intake with 1000 mg/day is recommended for achieving neutral calcium balance [31]. Additional calcium supplements or calcium-containing medications should be avoided for patients with adequate daily calcium intakes of 800–1000 mg per day [31].

Administration of a calcimimetic agent increases the sensitivity of CaSR and vitamin D receptor (VDR) expression [95], decreases PTH gene expression [96] and PTH secretion of the parathyroid gland [97]. In patients with CKD G5D, calcimimetics, such as cinacalcet, is suggested to increase BMD, normalize bone histology and reduce risk of fractures when serum PTH remain elevated despite sufficient calcidiol levels [89]. The Evaluation of Cinacalcet HCl Therapy to Lower Cardiovascular Events (EVOLVE) trial demonstrated that using cinacalcet for ≤64 months reduces the rate of clinical fracture by 16–29% [98], decreases serum FGF-23 and its-associated cardiovascular death and major cardiovascular events [99] in HD patients with SHPT. Treatment with cinacalcet for 1 year increases the BMD of the femoral neck in patients with iPTH level >300 pg/mL and undergoing HD, especially in those who had higher baseline serum bone-specific alkaline phosphatase (bALP) [100]. In the multicenter Bone Biopsy Study for Dialysis Patients with the Secondary Hyperparathyroidism of End Stage Renal Disease (BONAFIDE) study, long-term treatment with cinacalcet substantially reduced PTH, diminished the elevated bone formation rate, lowered several biochemical markers of high-turnover bone disease toward normal, and generally improved bone histology [101].

In a prospective trial of 41 patients with PD dialysis and biopsy-proven adynamic bone disease, low-calcium dialysate reduced serum calcium levels and hypercalcemic episodes, resulting in increased PTH levels and normalization of bone turnover [102]. Repeated bone biopsy after 16 months found that low-calcium dialysate led to normalization of bone formation rates and a 300% increase in PTH levels.

In patients after KT, calcium levels usually decrease early and elevate at three to six months post-transplantation [103]. Cinacalcet reduced hypercalcemia due to hyperparathyroidism [104]. However, a recent randomized study showed that subtotal parathyroidectomy was superior to cinacalcet in controlling hypercalcemia in patients with KT and tertiary hyperparathyroidism [105]. Cinacalcet is currently not approved for use in KT recipients [103]. Calcium supplementation alone is seldom required post-transplantation [103].

#### 5.3.3. Vitamin D

Synthesis of vitamin D is mainly reduced in CKD-MBD. Both 25(OH)D (calcidiol) and 1,25(OH)_2_D (calcitriol) play a crucial role on bone metabolism. Through vitamin D receptor (VDR) on bone cells, vitamin D stimulates calcium resorption and osteoclast differentiation via induction of Receptor activator of nuclear factor kappa B ligand (RANK-L) synthesis [17]. Nevertheless, the optimal circulating calcidiol and calcitriol levels to be considered remain unknown and controversial [106]. For CKD patients not on dialysis, the 2017 KDIGO guidelines recommend using 30 ng/mL cutoff value [40], which is the same as that used for the general population. For patients at CKD G5D, applying the threshold resulted in an estimated prevalence of vitamin D insufficiency (15–30 ng/mL) and/or deficiency (<15 ng/mL) ranging from 50% to 98% in a total sample size of 3722 HD patients [107]. Vitamin D supplementation is suggested to prescribe early in the process of CKD, but how to appropriately use it is still under debate [90,108]. Experts from Kidney Disease Outcomes Quality Initiative (KDOQI) and KDIGO have recognized that vitamin D insufficiency and deficiency should be avoided in CKD and dialysis patients by using supplementation to prevent SHPT [108]. Cholecalciferol (Vitamin D3) 800 IU/day is recommended for the treatment and prevention of vitamin D deficiency in CKD and dialysis patients [109]. Vitamin D provided during dialysis is more effective than home prescriptions [110]. The effects of vitamin D supplementation on CKD and dialysis patients include decreased serum PTH level, increased serum calcitriol level, reduced proteinuria, endothelial cardiovascular markers improvement and decreased inflammation markers [108].

In patients with CKD G3-4, both calcitriol and paricalcitol effectively decrease PTH and alkaline phosphatase levels with minimal effect on calcium levels and phosphorus balance [111]. Another small randomized trial of bone biopsy-proved adynamic bone disease showed that calcitriol decreased bone turnover [112], leading to the notion about decreasing active vitamin D therapy in these patients. Studies for non-dialysis CKD had shown that cholecalciferol (vitamin D3) may be superior to ergocalciferol (vitamin D2) in raising serum calcidiol for treating nutritional vitamin D deficiency [113,114]. In addition, a meta-analysis of three randomized control studies in patients with CKD G3-4 and hypovitaminosis D showed that supplementation of active/native vitamin D (cholecalciferol) did not show improvement in cardiac function and structure as calcitriol [115].

In CKD G5D, supplementation of calcidiol improves bone mineralization but has limited effect on reducing serum PTH level, while administration of calcitriol efficiently reduces serum PTH level but has little effect on bone mineralization [70]. Currently, there is strong evidence supporting calcidiol supplementation, aiming to control SHPT in CKD patients [116]. Despite the emerging observational data showing the association between lower levels of calcidiol and several deleterious outcomes (such as low bone turnover, risk of falls and fractures, progression of CKD and mortality) [70], there is still a lack of randomized control trials to support the potential beneficial effects of vitamin D supplementation.

In KT recipients with CKD-MBD, low serum calcidiol levels are common. Administration of calcitriol could decrease PTH after transplantation and increase femoral neck and lumbar spine BMD. However, there is no clear evidence in reducing fracture risk [117].

#### 5.3.4. Parathyroidectomy

In a national cohort of long-term CKD G5D patients, parathyroidectomy reduced bone turnover and improved BMD and risk of fractures [118].

### 5.4. Choices of Pharmacologic Treatment

**Figure 3:**
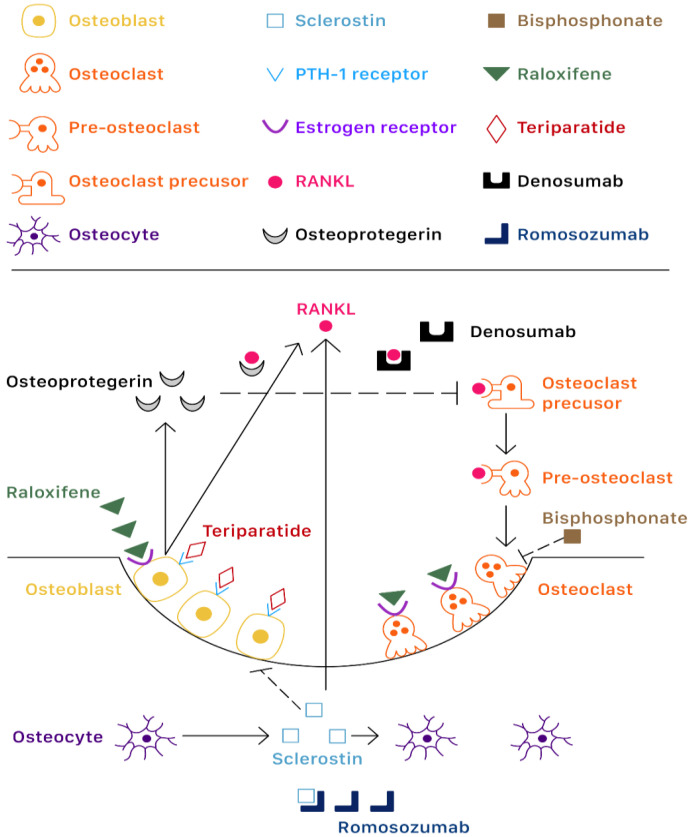
Bone remodeling and effects of bisphosphonate, raloxifene, teriparatide, denosumab, and romosozumab on bone. Normally bone is continuously renewed through bone formation by osteoblasts and bone resorption by osteoclasts. Osteoblasts secret receptor activator of NF-κB ligand (RANK-L) and osteoprotegerin, which are main factors of bone remodeling. RANK-L stimulate the differentiation of osteoclast precusors and pre-osteoclasts through RANK-L and M-CSF receptors. Osteoprotegerin is a soluble combining inhibitor of RANK-L, which could reduce osteoclasts differentiation. Osteocytes secret sclerostin, which could reduce osteoblastogenesis and stimulate osteoclastogenesis by inducing RANK-L synthesis. Bisphosphonates have high affinity to hydroxyapatite and selectively inhibit farnesyl pyrophosphate synthase within osteoclasts and induce osteoclast apoptosis. Raloxifene is a selective estrogen receptor modulator and could enhance osteoclast apoptosis [119]. Intermittent administration of teriparatide could stimulate bone formation through the PTH-1 receptor expressed only in osteoblasts. Denosumab targets RANK-L, leading to suppression of osteoclasts differentiation. Romosozumab binds sclerostin, leading to both osteoblast activation and reduced osteoclastogenesis.

There are a variety of anti-osteoporotic drugs which can be considered as pharmacologic treatment for osteoporosis. The actions and mechanisms of current anti-osteoporotic agents in CKD patients are showed in Figure 3.

#### 5.4.1. Antiresorptive

##### Bisphosphonates

Bisphosphonates are chemically stable derivatives of inorganic pyrophosphate that have a high affinity to hydroxyapatite crystals [120]. Nitrogen-containing bisphosphonates, such as zoledronic acid, risedronate, ibandronate and alendronate, selectively inhibit farnesyl pyrophosphate synthase within osteoclasts and induce osteoclast apoptosis [120,121], causing an overall reduction on bone absorption by decreasing osteoclast activity. The bisphosphonates are cleared by the kidney and retained in bone. Because of the unknown implications of greater bone retention of patients with CKD, the US FDA contradicted bisphosphonates exposure in those who have a creatinine clearance <35 mL/min.

In patients with CKD G1–4, a retrospective analysis that pooled nine clinical trials showed that risedronate increased BMD and prevented vertebral fractures regardless of degree of renal impairment [122]. In patients with CKD G1–3, a post hoc analysis of three Japanese trials concluded that risedronate could increase lumbar spine BMD without differences in CKD stage 1-3 [123]. In a secondary analysis of the Fracture Intervention Trial (FIT), administration of alendronate was safe and effective in increasing total hip and BMD and reducing spinal fractures in women with eGFR <45 mL/min [124]. Although there was a small increase in creatinine during the three-year study, the increase was similar to those without renal function impairment. In FIT trial, women with serum creatinine >1.27 mg/dL, serum PTH >85 pg/mL in isolation, or serum PTH >65 pg/mL in combination with abnormal serum calcium, alkaline phosphatase, or phosphate were excluded. In CKD G3–4, administration of weekly alendronate did not decrease the progression of vascular calcification compared with placebo, which was different from previous studies of HD patients [125]. There are insufficient data concerning efficacy of oral bisphosphonates in fracture prevention of patients with CKD-MBD and end-stage renal failure.

The zoledronic acid (Health Outcomes and Reduced Incidence with Zoledronic Acid Once Yearly, HORIZON) trials excluded subjects with CKD G4–5 [126]. Thus, there are too few patients in the zoledronic acid postmenopausal trials to generate data in CKD G4-5. In patients with CKD-MBD undergoing HD, ibandronate significantly increased BMD and decreased bone turnover [127]. In KT recipients, meta-analyses confirmed that bisphosphonates improved the femoral neck and lumbar spine BMD [128,129,130], but their effects on fracture risk and safety among KT recipients are not established [128].

##### Denosumab

Denosumab is a fully human monoclonal immunoglobulin (Ig)G2 antibody which targets and binds to the DE loop region of RANK-L with high affinity and specificity [131]. RANK-L signaling via RANK is the main factor for osteoclastogenesis and osteoclast activation [132]. RANK-L is also expressed by T-helper cells and involved in dentritic cell maturation [17]. RANK-L knockout mice had been demonstrated to develop severe osteoporosis as well as defective T and B lymphocytes differentiation [133]. Denosumab inhibits osteoclast proliferation and development, making it a potent anti-resorptive agent [8]. Evidence of safety and efficacy of using denosumab in preventing osteoporotic vertebral, non-vertebral, and hip fractures is sufficient in general population [134,135,136,137]. It does not increase the risk of aortic calcification progression or cardiovascular events in post-menopausal women [138]. A pharmacokinetic and pharmacodynamic study of single dose denosumab in patients with CKD or dialysis showed that serum concentration of denosumab did not differ in different degrees of renal insufficiency [139]. Furthermore, the effect of denosumab on increasing BMD and reducing fracture did not differ depending on renal function [140]. Because it is cleared by the reticuloendothelial system and not by the kidney, there is no restriction of its use in patients with eGFR <35 mL/min [89].

In a post hoc analysis of the FREEDOM (Fracture Reduction Evaluation of Denosumab in Osteoporosis Every 6 Months) trial, denosumab for 36 months effectively reduced vertebral, hip, and nonvertebral fracture risks without an increase in adverse events among postmenopausal women with CKD G1–4 [140]. However, there are insufficient data about fracture prevention in advance CKD because there were only 73 women with CKD G4 and no women with CKD G5. After previous bisphosphonate therapy, denosumab could increase BMD and is comparable to zoledronate in CKD patients [141]. In a small study in CKD G5D patients with SHPT and high-turnover bone disease, administration of denosumab resulted in an increase in femoral neck and lumbar spine BMD at 6 months [142]. Another small retrospective study also demonstrated the safety and efficacy of denosumab in the treatment of osteoporosis in CKD G5D patients [143]. Administration of denosumab is associated with a significant risk of hypocalcemia in patients with CKD [139,144,145] and KT [146]. In kidney transplant recipients, denosumab could improve hypercalcemia and BMD loss [147]. A recent systemic review and meta-analysis study showed that denosumab could effectively increase BMD and T scores in the lumbar spine and femur neck among KT recipients with good allograft function [148]. Moreover, a retrospective head-to-head study showed that the effect of denosumab in improving lumbar spine and femoral neck BMD was greater compared with that of bisphosphonate treatment [149]. Denosumab treatment should not be discontinued post KT because discontinuation of the treatment would lead to an increased risk of vertebral fractures and rapid loss of BMD [150]. Close monitoring and supplement of calcium and calcitriol preemptively before starting denosumab are needed to avoid the side effect of hypocalcemia [151,152,153]. In addition, CKD G4–5 and male sex were found to be associated with denosumab-induced hypocalcemia [154]. It is not rare that worsening SHPT (intact PTH levels rise greater than 1000 pg/mL) under denosumab treatment, even with concomitant administration of massive active vitamin D [155]. Further studies are needed for its safety in efficacy of fracture prevention in patients with advanced CKD and post kidney transplantation bone loss [103].

##### Raloxifene

Raloxifene is a selective estrogen receptor modulator (SERM) approved by FDA for prevention and treatment of osteoporosis in postmenopausal women [156]. It manifests estrogenic activity in bone, and in cardiovascular systems while opposing estrogen action in the uterus and breast [157], thus displaying less risk of invasive breast cancers compared with estrogens.

In a post hoc analysis of the Multiple Outcomes of Raloxifene Evaluation (MORE) trial, raloxifene improved BMD and reduced vertebral fractures in patients with postmenopausal osteoporosis and CKD G1–4, irrespective of kidney function [158]. In CKD G5, raloxifene has efficacy of improving BMD without adverse effects and significant effect on controlling hyperparathyroidism [159]. In CKD G5D postmenopausal women on HD, raloxifene significantly increases trabecular BMD, decreases bone resorption markers and LDL-cholesterol values after 1-year of treatment [160]. Larger clinical trials are needed to determine its efficacy and safety on bone in patients with CKD-MBD after KT.

#### 5.4.2. Anabolic Agents

##### Teriparatide

Teriparatide is a recombinant peptide of the first 34 amino-*N*-terminal residues of PTH. It is an effective FDA-approved osteoanabolic drug that increases BMD and reduces fracture risk in both age-related and glucocorticoid-induced osteoporosis. Trabecular bone increases more than cortical bone [161]. The anabolic effect of intermittent PTH on bone is likely to be mediated through the PTH-1 receptor [162], which is selective for the *N*-terminal region of the molecule [163]. PTH-1 receptor is expressed in osteoblast but not in osteoclast [17]. PTH1-receptor activation increases osteoblasts’ number and activity initially and leads to new osteoclasts recruitment later via increasing RANK-L synthesis and decreasing osteoprotegerin (OPG) synthesis. Furthermore, PTH inhibits sclerostin binding to osteoblasts, which results in promoting bone formation [164,165]. Its stimulation of bone formation occurs earlier than bone resorption [17]. PTH effects are not persistent after discontinuation of therapy, unless anti-resorptive agents are given [166].

There are no sufficient clinical data examining the efficacy of teriparatide in patients with CKD-MBD because most clinical trials excluded patients with elevated baseline serum levels of PTH. In a post hoc analysis of the Fracture Prevention Trial, teriparatide increases lumbar and femoral neck BMD in female patients with post-menopausal osteoporosis and eGFR as low as 30 mL/min [167]. Another post hoc analysis of a post marketing surveillance study, including thirty patients with CKD G4 and three patients with CKD G5, showed that daily teriparatide improved BMD and incidences of new vertebral and nonvertebral fractures in patients with osteoporosis with high risk of fracture [168]. Some data of using teriparatide on patients with CKD-MBD is available from small observational studies. In patients with CKD G5D and biopsy proven adynamic bone disease, daily administration of teriparatide for 6 months resulted in lumbar spine and femoral neck BMD [169]. Increased bone turnover after using teriparatide has also been found in some CKD G5D cases [170,171]. In patients with CKD G5D with hypothyroidism and osteoporosis, administration of teriparatide once a week increased lumbar spine BMD, bone formation, and resorption markers [172]. Conversely, 6-month daily subcutaneous injections of 20 μg teriparatide did not improve BMD early after KT in a case series [173]. Larger clinical trials are needed to determine the efficacy and safety of teriparatide in the patients with CKD-MBD, undergoing dialysis and post KT.

##### Abaloparatide

Abaloparatide is a recombinant peptide of the first 20 amino-*N*-terminal residues of PTH. It was designed to have relatively greater transient affinity of PTH/PTH1 receptor [174] and be more purely anabolic. Although it has been proven for its efficacy in improving lumbar spine and total hip BMD in postmenopausal women with osteoporosis [175,176], there are no available data concerning abaloparatide in patients with CKD-MBD. The ability of abaloparatide to increase bone mass and formation with less risk of hypercalcemia than teriparatide makes it become an ideal drug to treat patients with CKD-MBD and low-to-normal bone turnover with high fracture risk [175].

##### Romosozumab

Romosozumab is a humanized monoclonal IgG2 anti-sclerostin antibody [17]. It targets and binds sclerostin, a Wnt antagonist produced by osteocytes that reduces osteoblastogenesis and promotes osteoclastogenesis [177]. This monoclonal antibody against sclerostin increases bone formation and decreases bone resorption [17,178]. Although clinical studies with romosozumab have shown to increase bone density and reduce vertebral and nonvertebral fractures in postmenopausal women [178,179,180], the precise role and its action in CKD patients remain unknown [90].

Sclerostin may promote vascular calcification [177]. Extra-skeletal effects, such as vascular calcification should be cautious when dealing with romosozumab. Because of the activity of Wnt signaling influencing the integrity of the arterial wall [181], blocking sclerostin will impact the vascular calcification processes. It has not been elucidated how anti-sclerostin antibody affect vascular calcifications [8,17] and cardiovascular mortality in CKD patients [23].

## 6. Discussion

Changes in mineral and humoral metabolism as well as bone structure develop early in the course of CKD. CKD-MBD included abnormalities of calcium, phosphorus, PTH, and/or vitamin D; abnormalities in bone turnover, mineralization, volume, linear growth, or strength; and/or vascular or other soft tissue calcification [2]. In patients with CKD-MBD, using DXA or FRAX to screen fracture risk should be considered. Biomarkers such as bALP and iPTH may assist to assess bone turnover, except for bone biopsy [8]. Among CKD patients, adynamic bone disease or low bone turnover is the most common type of renal osteodystrophy. Before initiating an antiresorptive or anabolic agent to treat osteoporosis in CKD patients, lifestyle modifications, such as exercise, calcium, vitamin D supplementation, smoking cessation, and avoidance of excessive alcohol intake are important. Managing hyperphosphatemia and SHPT are also crucial. Non-calcium-based phosphate binders, such as sevelamer, are superior to calcium-based binder in increasing the bone formation rate and improving trabecular architecture [43]. In patients with CKD G1–3, physicians should use bisphosphonates and other osteoporosis treatments the same as for patients without CKD. Bisphosphonates are generally not recommended in patients with eGFR <35 mL/min or evidence of adynamic bone disease due to avoiding over-suppression of bone remodeling. In KT recipients, bisphosphonates have efficacy in improving femoral neck and lumbar spine BMD [128,129,130]. Denosumab can be safely administered to CKD-associated osteoporosis, but its side effect of hypocalcemia should be cautious. Data concerning using anabolic agents in patients with CKD-MBD are limited. Larger studies are needed to assess the role of teriparatide or abaloparatide in adynamic bone disease. Since mounting evidence points to a central role of a disturbed Wnt–β-catenin signaling in the pathogenesis of CKD-MBD and its cross-talk between the kidneys, the vasculature, and the bone [16], targeting Wnt inhibitors, such as sclerostin or dickkopf1, by monoclonal antibodies would be a potential choice to treat osteoporosis in CKD-MBD. Studies evaluating the optimal diagnostic and management strategy in patients with CKD G4–5D are needed.

## 7. Conclusions

Understanding the complex pathogenesis of CKD-MBD is crucial in helping us to improve one’s short- and long-term outcomes. Treatment strategies for CKD-associated osteoporosis should be patient-centered to determine the type of renal osteodystrophy. This review focuses on the mechanism, evaluation and management of patients with CKD-MBD. However, further studies are needed to explore more details regarding the underlying pathophysiology and to assess the safety and efficacy of agents for treating CKD-MBD.

## Figures and Tables

**Table 1:** Factors affecting bone strength in chronic kidney disease-mineral and bone disorder (CKD-MBD).

Factor	Main Effect	Category
↓ Kloth	↑ FGF-23 level [13]	Humoral
↑ FGF-23 ^1^	↑ phosphate excretion [14] ↓ calcitriol synthesis [14,15]	Humoral
↑ Sclerostin	↓ bone formation [14,16] ↑ osteoclastogenesis [17]	Humoral
↑ dickkopf1	↓ bone formation [16,18]	Humoral
↑ phosphate	↑ SPTH ^3^ [19] ↓ calcitriol synthesis [20]	Mineral
↑ uremic toxins ^2^	↓ PTH receptor [21] ↑ skeletal resistance to PTH [21]	Uremia
↓ 1,25(OH)_2_ D	↑ PTH secretion [20,22] ↓ calcium [23]	Humoral
↓ calcium	↑ SPTH [23] ↑ abnormal bone remodeling [23]	Mineral
↑Skeletal resistance to PTH	↑ SPTH [24]	Humoral

^1^ FGF-23: fibroblast growth factor-23; ^2^ Uremic toxins refer to indoxyl sulfate and p-cresyl sulfate; ^3^ SPTH: secondary hyperparathyroidism; ↓: decrease, ↑: increase

## References

[1] Kidney Disease: Improving Global Outcomes (KDIGO) CKD-MBD Update Work Group (2017). KDIGO 2017 Clinical Practice Guideline Update for the Diagnosis, Evaluation, Prevention, and Treatment of Chronic Kidney Disease-Mineral and Bone Disorder (CKD-MBD). Kidney Int. Suppl..

[2] Moe S., Drueke T., Cunningham J., Goodman W., Martin K., Olgaard K., Ott S., Sprague S., Lameire N., Eknoyan G. (2006). Definition, evaluation, and classification of renal osteodystrophy: A position statement from Kidney Disease: Improving Global Outcomes (KDIGO). Kidney Int..

[3] Sidibe A., Auguste D., Desbiens L.C., Fortier C., Wang Y.P., Jean S., Moore L., Mac-Way F. (2019). Fracture Risk in Dialysis and Kidney Transplanted Patients: A Systematic Review. JBMR Plus.

[4] Nih Consensus Development Panel on Osteoporosis Prevention, Diagnosis, and Therapy (2001). Osteoporosis prevention, diagnosis, and therapy. JAMA.

[5] Najar M.S., Mir M.M., Muzamil M. (2017). Prevalence of osteoporosis in patients with chronic kidney disease (stages 3–5) in comparison with age- and sex-matched controls: A study from Kashmir Valley Tertiary Care Center. Saudi J. Kidney Dis. Transplant..

[6] Nickolas T.L., McMahon D.J., Shane E. (2006). Relationship between moderate to severe kidney disease and hip fracture in the United States. J. Am. Soc. Nephrol..

[7] Bezerra de Carvalho K.S., Vasco R.F.V., Custodio M.R., Jorgetti V., Moyses R.M.A., Elias R.M. (2019). Chronic kidney disease is associated with low BMD at the hip but not at the spine. Osteoporos. Int..

[8] Khairallah P., Nickolas T.L. (2018). Management of Osteoporosis in CKD. Clin. J. Am. Soc. Nephrol..

[9] Kim S.M., Long J., Montez-Rath M., Leonard M., Chertow G.M. (2016). Hip Fracture in Patients with Non-Dialysis-Requiring Chronic Kidney Disease. J. Bone Miner. Res..

[10] Chen H., Lips P., Vervloet M.G., van Schoor N.M., de Jongh R.T. (2018). Association of renal function with bone mineral density and fracture risk in the Longitudinal Aging Study Amsterdam. Osteoporos. Int..

[11] Hall R.K., Sloane R., Pieper C., Van Houtven C., LaFleur J., Adler R., Colon-Emeric C. (2018). Competing Risks of Fracture and Death in Older Adults with Chronic Kidney Disease. J. Am. Geriatr. Soc..

[12] Moe S.M. (2017). Renal Osteodystrophy or Kidney-Induced Osteoporosis?. Curr. Osteoporos. Rep..

[13] Hu M.C., Shi M., Zhang J., Quinones H., Griffith C., Kuro-o M., Moe O.W. (2011). Klotho deficiency causes vascular calcification in chronic kidney disease. J. Am. Soc. Nephrol..

[14] Mazzaferro S., Cianciolo G., De Pascalis A., Guglielmo C., Urena Torres P.A., Bover J., Tartaglione L., Pasquali M., La Manna G. (2018). Bone, inflammation and the bone marrow niche in chronic kidney disease: What do we know?. Nephrol. Dial. Transplant..

[15] Gutierrez O., Isakova T., Rhee E., Shah A., Holmes J., Collerone G., Juppner H., Wolf M. (2005). Fibroblast growth factor-23 mitigates hyperphosphatemia but accentuates calcitriol deficiency in chronic kidney disease. J. Am. Soc. Nephrol..

[16] Evenepoel P., D’Haese P., Brandenburg V. (2015). Sclerostin and DKK1: New players in renal bone and vascular disease. Kidney Int..

[17] Tartaglione L., Pasquali M., Rotondi S., Muci M.L., Covic A., Mazzaferro S. (2017). Positioning novel biologicals in CKD-mineral and bone disorders. J. Nephrol..

[18] Colditz J., Thiele S., Baschant U., Garbe A.I., Niehrs C., Hofbauer L.C., Rauner M. (2019). Osteogenic Dkk1 Mediates Glucocorticoid-Induced but Not Arthritis-Induced Bone Loss. J. Bone Miner. Res..

[19] Silver J., Levi R. (2005). Cellular and molecular mechanisms of secondary hyperparathyroidism. Clin. Nephrol..

[20] Llach F. (1995). Secondary hyperparathyroidism in renal failure: The trade-off hypothesis revisited. Am. J. Kidney Dis..

[21] Yamamoto S., Fukagawa M. (2017). Uremic Toxicity and Bone in CKD. J. Nephrol..

[22] Malluche H.H., Mawad H., Koszewski N.J. (2002). Update on vitamin D and its newer analogues: Actions and rationale for treatment in chronic renal failure. Kidney Int..

[23] Iwasaki Y., Kazama J.J., Fukagawa M. (2017). Molecular Abnormalities Underlying Bone Fragility in Chronic Kidney Disease. Biomed Res. Int..

[24] Naveh-Many T., Rahamimov R., Livni N., Silver J. (1995). Parathyroid cell proliferation in normal and chronic renal failure rats. The effects of calcium, phosphate, and vitamin D. J. Clin. Investig..

[25] Levin A., Bakris G.L., Molitch M., Smulders M., Tian J., Williams L.A., Andress D.L. (2007). Prevalence of abnormal serum vitamin D, PTH, calcium, and phosphorus in patients with chronic kidney disease: Results of the study to evaluate early kidney disease. Kidney Int..

[26] Rodriguez M., Nemeth E., Martin D. (2005). The calcium-sensing receptor: A key factor in the pathogenesis of secondary hyperparathyroidism. Am. J. Physiol. Renal Physiol..

[27] Canadillas S., Canalejo A., Santamaria R., Rodriguez M.E., Estepa J.C., Martin-Malo A., Bravo J., Ramos B., Aguilera-Tejero E., Rodriguez M. (2005). Calcium-sensing receptor expression and parathyroid hormone secretion in hyperplastic parathyroid glands from humans. J. Am. Soc. Nephrol..

[28] Block G.A., Hulbert-Shearon T.E., Levin N.W., Port F.K. (1998). Association of serum phosphorus and calcium x phosphate product with mortality risk in chronic hemodialysis patients: A national study. Am. J. Kidney Dis..

[29] Floege J., Kim J., Ireland E., Chazot C., Drueke T., de Francisco A., Kronenberg F., Marcelli D., Passlick-Deetjen J., Schernthaner G. (2011). Serum iPTH, calcium and phosphate, and the risk of mortality in a European haemodialysis population. Nephrol. Dial. Transplant..

[30] Sessa A., Esposito A., Iavicoli G.D., Lettieri E., Dente G., Costa C., Bergallo M., Rossano R., Capuano M. (2010). Immunosuppressive agents and bone disease in renal transplant patients with hypercalcemia. Transplant. Proc..

[31] Hill Gallant K.M., Spiegel D.M. (2017). Calcium Balance in Chronic Kidney Disease. Curr. Osteoporos. Rep..

[32] Martin K.J., Gonzalez E.A. (2007). Metabolic bone disease in chronic kidney disease. J. Am. Soc. Nephrol..

[33] Hruska K.A., Teitelbaum S.L. (1995). Renal osteodystrophy. N. Engl. J. Med..

[34] Saito H., Maeda A., Ohtomo S., Hirata M., Kusano K., Kato S., Ogata E., Segawa H., Miyamoto K., Fukushima N. (2005). Circulating FGF-23 is regulated by 1alpha,25-dihydroxyvitamin D3 and phosphorus in vivo. J. Biol. Chem..

[35] Paloian N.J., Giachelli C.M. (2014). A current understanding of vascular calcification in CKD. Am. J. Physiol. Renal Physiol..

[36] Isakova T., Wahl P., Vargas G.S., Gutierrez O.M., Scialla J., Xie H., Appleby D., Nessel L., Bellovich K., Chen J. (2011). Fibroblast growth factor 23 is elevated before parathyroid hormone and phosphate in chronic kidney disease. Kidney Int..

[37] Ross A.C., Manson J.E., Abrams S.A., Aloia J.F., Brannon P.M., Clinton S.K., Durazo-Arvizu R.A., Gallagher J.C., Gallo R.L., Jones G. (2011). The 2011 report on dietary reference intakes for calcium and vitamin D from the Institute of Medicine: What clinicians need to know. J. Clin. Endocrinol. Metab..

[38] Lips P., Cashman K.D., Lamberg-Allardt C., Bischoff-Ferrari H.A., Obermayer-Pietsch B., Bianchi M.L., Stepan J., El-Hajj Fuleihan G., Bouillon R. (2019). Current vitamin D status in European and Middle East countries and strategies to prevent vitamin D deficiency: A position statement of the European Calcified Tissue Society. Eur. J. Endocrinol..

[39] LaClair R.E., Hellman R.N., Karp S.L., Kraus M., Ofner S., Li Q., Graves K.L., Moe S.M. (2005). Prevalence of calcidiol deficiency in CKD: A cross-sectional study across latitudes in the United States. Am. J. Kidney Dis..

[40] Coen G., Mantella D., Manni M., Balducci A., Nofroni I., Sardella D., Ballanti P., Bonucci E. (2005). 25-hydroxyvitamin D levels and bone histomorphometry in hemodialysis renal osteodystrophy. Kidney Int..

[41] Jacome-Galarza C.E., Percin G.I., Muller J.T., Mass E., Lazarov T., Eitler J., Rauner M., Yadav V.K., Crozet L., Bohm M. (2019). Developmental origin, functional maintenance and genetic rescue of osteoclasts. Nature.

[42] Felsenberg D., Boonen S. (2005). The bone quality framework: Determinants of bone strength and their interrelationships, and implications for osteoporosis management. Clin. Ther..

[43] Ferreira A., Frazao J.M., Monier-Faugere M.C., Gil C., Galvao J., Oliveira C., Baldaia J., Rodrigues I., Santos C., Ribeiro S. (2008). Effects of sevelamer hydrochloride and calcium carbonate on renal osteodystrophy in hemodialysis patients. J. Am. Soc. Nephrol..

[44] Barreto F.C., Barreto D.V., Moyses R.M., Neves K.R., Canziani M.E., Draibe S.A., Jorgetti V., Carvalho A.B. (2008). K/DOQI-recommended intact PTH levels do not prevent low-turnover bone disease in hemodialysis patients. Kidney Int..

[45] Spasovski G.B., Bervoets A.R., Behets G.J., Ivanovski N., Sikole A., Dams G., Couttenye M.M., De Broe M.E., D’Haese P.C. (2003). Spectrum of renal bone disease in end-stage renal failure patients not yet on dialysis. Nephrol. Dial. Transplant..

[46] Sprague S.M., Bellorin-Font E., Jorgetti V., Carvalho A.B., Malluche H.H., Ferreira A., D’Haese P.C., Drueke T.B., Du H., Manley T. (2016). Diagnostic Accuracy of Bone Turnover Markers and Bone Histology in Patients With CKD Treated by Dialysis. Am. J. Kidney Dis..

[47] Malluche H.H., Mawad H.W., Monier-Faugere M.C. (2011). Renal osteodystrophy in the first decade of the new millennium: Analysis of 630 bone biopsies in black and white patients. J. Bone Miner. Res..

[48] Moe S.M., Drueke T.B. (2004). A bridge to improving healthcare outcomes and quality of life. Am. J. Kidney Dis..

[49] Barreto F.C., Barreto D.V., Moyses R.M., Neves C.L., Jorgetti V., Draibe S.A., Canziani M.E., Carvalho A.B. (2006). Osteoporosis in hemodialysis patients revisited by bone histomorphometry: A new insight into an old problem. Kidney Int..

[50] Gal-Moscovici A., Popovtzer M.M. (2005). New worldwide trends in presentation of renal osteodystrophy and its relationship to parathyroid hormone levels. Clin. Nephrol..

[51] Wehrli F.W., Leonard M.B., Saha P.K., Gomberg B.R. (2004). Quantitative high-resolution magnetic resonance imaging reveals structural implications of renal osteodystrophy on trabecular and cortical bone. J. Magn. Reson. Imaging.

[52] Qunibi W., Moustafa M., Muenz L.R., He D.Y., Kessler P.D., Diaz-Buxo J.A., Budoff M., CARE-2 Investigators (2008). A 1-year randomized trial of calcium acetate versus sevelamer on progression of coronary artery calcification in hemodialysis patients with comparable lipid control: The Calcium Acetate Renagel Evaluation-2 (CARE-2) study. Am. J. Kidney Dis..

[53] Leu H.J., Brunner U. (1992). Calcified and ossified phlebosclerosis. VASA.

[54] Vervloet M., Cozzolino M. (2017). Vascular calcification in chronic kidney disease: Different bricks in the wall?. Kidney Int..

[55] London G.M., Guerin A.P., Marchais S.J., Metivier F., Pannier B., Adda H. (2003). Arterial media calcification in end-stage renal disease: Impact on all-cause and cardiovascular mortality. Nephrol. Dial. Transplant..

[56] Goodman W.G., Goldin J., Kuizon B.D., Yoon C., Gales B., Sider D., Wang Y., Chung J., Emerick A., Greaser L. (2000). Coronary-artery calcification in young adults with end-stage renal disease who are undergoing dialysis. N. Engl. J. Med..

[57] Goldsmith D.J., Covic A., Sambrook P.A., Ackrill P. (1997). Vascular calcification in long-term haemodialysis patients in a single unit: A retrospective analysis. Nephron.

[58] McCullough P.A., Sandberg K.R., Dumler F., Yanez J.E. (2004). Determinants of coronary vascular calcification in patients with chronic kidney disease and end-stage renal disease: A systematic review. J. Nephrol..

[59] Ok E., Asci G., Bayraktaroglu S., Toz H., Ozkahya M., Yilmaz M., Kircelli F., Sevinc Ok E., Ceylan N., Duman S. (2016). Reduction of Dialysate Calcium Level Reduces Progression of Coronary Artery Calcification and Improves Low Bone Turnover in Patients on Hemodialysis. J. Am. Soc. Nephrol..

[60] Ishimura E., Okuno S., Kitatani K., Tsuchida T., Yamakawa T., Shioi A., Inaba M., Nishizawa Y. (2007). Significant association between the presence of peripheral vascular calcification and lower serum magnesium in hemodialysis patients. Clin. Nephrol..

[61] Kramer H., Toto R., Peshock R., Cooper R., Victor R. (2005). Association between chronic kidney disease and coronary artery calcification: The Dallas Heart Study. J. Am. Soc. Nephrol..

[62] Han K.H., O’Neill W.C. (2016). Increased Peripheral Arterial Calcification in Patients Receiving Warfarin. J. Am. Heart Assoc..

[63] Wada K., Wada Y. (2014). Evaluation of aortic calcification with lanthanum carbonate vs. calcium-based phosphate binders in maintenance hemodialysis patients with type 2 diabetes mellitus: An open-label randomized controlled trial. Ther. Apher. Dial..

[64] Raggi P., Chertow G.M., Torres P.U., Csiky B., Naso A., Nossuli K., Moustafa M., Goodman W.G., Lopez N., Downey G. (2011). The ADVANCE study: A randomized study to evaluate the effects of cinacalcet plus low-dose vitamin D on vascular calcification in patients on hemodialysis. Nephrol. Dial. Transplant..

[65] O’Neill W.C., Sigrist M.K., McIntyre C.W. (2010). Plasma pyrophosphate and vascular calcification in chronic kidney disease. Nephrol. Dial. Transplant..

[66] Collin-Osdoby P. (2004). Regulation of vascular calcification by osteoclast regulatory factors RANKL and osteoprotegerin. Circ. Res..

[67] West S.L., Lok C.E., Langsetmo L., Cheung A.M., Szabo E., Pearce D., Fusaro M., Wald R., Weinstein J., Jamal S.A. (2015). Bone mineral density predicts fractures in chronic kidney disease. J. Bone Miner. Res..

[68] Naylor K.L., Garg A.X., Zou G., Langsetmo L., Leslie W.D., Fraser L.A., Adachi J.D., Morin S., Goltzman D., Lentle B. (2015). Comparison of fracture risk prediction among individuals with reduced and normal kidney function. Clin. J. Am. Soc. Nephrol..

[69] Iimori S., Mori Y., Akita W., Kuyama T., Takada S., Asai T., Kuwahara M., Sasaki S., Tsukamoto Y. (2012). Diagnostic usefulness of bone mineral density and biochemical markers of bone turnover in predicting fracture in CKD stage 5D patients—A single-center cohort study. Nephrol. Dial. Transplant..

[70] Torres P.A.U., Cohen-Solal M. (2017). Evaluation of fracture risk in chronic kidney disease. J. Nephrol..

[71] Jamal S.A., West S.L., Nickolas T.L. (2014). The clinical utility of FRAX to discriminate fracture status in men and women with chronic kidney disease. Osteoporos. Int..

[72] Naylor K.L., Leslie W.D., Hodsman A.B., Rush D.N., Garg A.X. (2014). FRAX predicts fracture risk in kidney transplant recipients. Transplantation.

[73] Imanishi Y., Inaba M., Nakatsuka K., Nagasue K., Okuno S., Yoshihara A., Miura M., Miyauchi A., Kobayashi K., Miki T. (2004). FGF-23 in patients with end-stage renal disease on hemodialysis. Kidney Int..

[74] Larsson T., Nisbeth U., Ljunggren O., Juppner H., Jonsson K.B. (2003). Circulating concentration of FGF-23 increases as renal function declines in patients with chronic kidney disease, but does not change in response to variation in phosphate intake in healthy volunteers. Kidney Int..

[75] De Seigneux S., Courbebaisse M., Rutkowski J.M., Wilhelm-Bals A., Metzger M., Khodo S.N., Hasler U., Chehade H., Dizin E., Daryadel A. (2015). Proteinuria Increases Plasma Phosphate by Altering Its Tubular Handling. J. Am. Soc. Nephrol..

[76] Goyal R., Jialal I. (2020). Hyperphosphatemia. StatPearls.

[77] Fournier A., Moriniere P., Ben Hamida F., el Esjer N., Shenovda M., Ghazali A., Bouzernidj M., Achard J.M., Westeel P.F. (1992). Use of alkaline calcium salts as phosphate binder in uremic patients. Kidney Int. Suppl..

[78] Komaba H., Goto S., Fujii H., Hamada Y., Kobayashi A., Shibuya K., Tominaga Y., Otsuki N., Nibu K., Nakagawa K. (2010). Depressed expression of Klotho and FGF receptor 1 in hyperplastic parathyroid glands from uremic patients. Kidney Int..

[79] Canalejo R., Canalejo A., Martinez-Moreno J.M., Rodriguez-Ortiz M.E., Estepa J.C., Mendoza F.J., Munoz-Castaneda J.R., Shalhoub V., Almaden Y., Rodriguez M. (2010). FGF23 fails to inhibit uremic parathyroid glands. J. Am. Soc. Nephrol..

[80] Hsu C.H., Patel S.R., Young E.W., Vanholder R. (1994). The biological action of calcitriol in renal failure. Kidney Int..

[81] Denda M., Finch J., Brown A.J., Nishii Y., Kubodera N., Slatopolsky E. (1996). 1,25-dihydroxyvitamin D3 and 22-oxacalcitriol prevent the decrease in vitamin D receptor content in the parathyroid glands of uremic rats. Kidney Int..

[82] Slatopolsky E., Weerts C., Thielan J., Horst R., Harter H., Martin K.J. (1984). Marked suppression of secondary hyperparathyroidism by intravenous administration of 1,25-dihydroxy-cholecalciferol in uremic patients. J. Clin. Investig..

[83] Wilson L., Felsenfeld A., Drezner M.K., Llach F. (1985). Altered divalent ion metabolism in early renal failure: Role of 1,25(OH)2D. Kidney Int..

[84] Yano S., Sugimoto T., Tsukamoto T., Chihara K., Kobayashi A., Kitazawa S., Maeda S., Kitazawa R. (2000). Association of decreased calcium-sensing receptor expression with proliferation of parathyroid cells in secondary hyperparathyroidism. Kidney Int..

[85] Gogusev J., Duchambon P., Hory B., Giovannini M., Goureau Y., Sarfati E., Drueke T.B. (1997). Depressed expression of calcium receptor in parathyroid gland tissue of patients with hyperparathyroidism. Kidney Int..

[86] Chen J., Zhang X., Zhang H., Liu T., Zhang H., Teng J., Ji J., Ding X. (2016). Indoxyl Sulfate Enhance the Hypermethylation of Klotho and Promote the Process of Vascular Calcification in Chronic Kidney Disease. Int. J. Biol. Sci..

[87] Tanaka H., Iwasaki Y., Yamato H., Mori Y., Komaba H., Watanabe H., Maruyama T., Fukagawa M. (2013). p-Cresyl sulfate induces osteoblast dysfunction through activating JNK and p38 MAPK pathways. Bone.

[88] West S.L., Jamal S.A., Lok C.E. (2012). Tests of neuromuscular function are associated with fractures in patients with chronic kidney disease. Nephrol. Dial. Transplant..

[89] Khairallah P., Nickolas T.L. (2018). Updates in CKD-Associated Osteoporosis. Curr. Osteoporos. Rep..

[90] Bover J., Bailone L., Lopez-Baez V., Benito S., Ciceri P., Galassi A., Cozzolino M. (2017). Osteoporosis, bone mineral density and CKD-MBD: Treatment considerations. J. Nephrol..

[91] Heiwe S., Jacobson S.H. (2014). Exercise training in adults with CKD: A systematic review and meta-analysis. Am. J. Kidney Dis..

[92] Liao H.W., Huang T.H., Chang Y.H., Liou H.H., Chou Y.H., Sue Y.M., Hung P.H., Chang Y.T., Ho P.C., Tsai K.J. (2019). Exercise Alleviates Osteoporosis in Rats with Mild Chronic Kidney Disease by Decreasing Sclerostin Production. Int. J. Mol. Sci..

[93] Roshanravan B., Gamboa J., Wilund K. (2017). Exercise and CKD: Skeletal Muscle Dysfunction and Practical Application of Exercise to Prevent and Treat Physical Impairments in CKD. Am. J. Kidney Dis..

[94] Hill K.M., Martin B.R., Wastney M.E., McCabe G.P., Moe S.M., Weaver C.M., Peacock M. (2013). Oral calcium carbonate affects calcium but not phosphorus balance in stage 3–4 chronic kidney disease. Kidney Int..

[95] Rodriguez M.E., Almaden Y., Canadillas S., Canalejo A., Siendones E., Lopez I., Aguilera-Tejero E., Martin D., Rodriguez M. (2007). The calcimimetic R-568 increases vitamin D receptor expression in rat parathyroid glands. Am. J. Physiol. Renal Physiol..

[96] Levi R., Ben-Dov I.Z., Lavi-Moshayoff V., Dinur M., Martin D., Naveh-Many T., Silver J. (2006). Increased parathyroid hormone gene expression in secondary hyperparathyroidism of experimental uremia is reversed by calcimimetics: Correlation with posttranslational modification of the trans acting factor AUF1. J. Am. Soc. Nephrol..

[97] Nagano N. (2006). Pharmacological and clinical properties of calcimimetics: Calcium receptor activators that afford an innovative approach to controlling hyperparathyroidism. Pharmacol. Ther..

[98] Moe S.M., Abdalla S., Chertow G.M., Parfrey P.S., Block G.A., Correa-Rotter R., Floege J., Herzog C.A., London G.M., Mahaffey K.W. (2015). Effects of Cinacalcet on Fracture Events in Patients Receiving Hemodialysis: The EVOLVE Trial. J. Am. Soc. Nephrol..

[99] Moe S.M., Chertow G.M., Parfrey P.S., Kubo Y., Block G.A., Correa-Rotter R., Drueke T.B., Herzog C.A., London G.M., Mahaffey K.W. (2015). Cinacalcet, Fibroblast Growth Factor-23, and Cardiovascular Disease in Hemodialysis: The Evaluation of Cinacalcet HCl Therapy to Lower Cardiovascular Events (EVOLVE) Trial. Circulation.

[100] Tsuruta Y., Okano K., Kikuchi K., Tsuruta Y., Akiba T., Nitta K. (2013). Effects of cinacalcet on bone mineral density and bone markers in hemodialysis patients with secondary hyperparathyroidism. Clin. Exp. Nephrol..

[101] Behets G.J., Spasovski G., Sterling L.R., Goodman W.G., Spiegel D.M., De Broe M.E., D’Haese P.C. (2015). Bone histomorphometry before and after long-term treatment with cinacalcet in dialysis patients with secondary hyperparathyroidism. Kidney Int..

[102] Haris A., Sherrard D.J., Hercz G. (2006). Reversal of adynamic bone disease by lowering of dialysate calcium. Kidney Int..

[103] Damasiewicz M.J., Ebeling P.R. (2017). Management of mineral and bone disorders in renal transplant recipients. Nephrology.

[104] Jo H.A., Han K.H., So Y.K., Jun H., Han S.Y. (2019). Effect of Cinacalcet in Kidney Transplant Patients with Hyperparathyroidism. Transplant. Proc..

[105] Cruzado J.M., Moreno P., Torregrosa J.V., Taco O., Mast R., Gomez-Vaquero C., Polo C., Revuelta I., Francos J., Torras J. (2016). A Randomized Study Comparing Parathyroidectomy with Cinacalcet for Treating Hypercalcemia in Kidney Allograft Recipients with Hyperparathyroidism. J. Am. Soc. Nephrol..

[106] Pimentel A., Urena-Torres P., Zillikens M.C., Bover J., Cohen-Solal M. (2017). Fractures in patients with CKD-diagnosis, treatment, and prevention: A review by members of the European Calcified Tissue Society and the European Renal Association of Nephrology Dialysis and Transplantation. Kidney Int..

[107] Singer R.F. (2013). Vitamin D in dialysis: Defining deficiency and rationale for supplementation. Semin. Dial..

[108] Jean G., Souberbielle J.C., Chazot C. (2017). Vitamin D in Chronic Kidney Disease and Dialysis Patients. Nutrients.

[109] Lips P., Goldsmith D., de Jongh R. (2017). Vitamin D and osteoporosis in chronic kidney disease. J. Nephrol..

[110] Delanaye P., Cavalier E., Fafin C., Dubois B.E., Krzesinski J.M., Moranne O. (2016). Efficiency of delivery observed treatment in hemodialysis patients: The example of the native vitamin D therapy. J. Nephrol..

[111] Coyne D.W., Goldberg S., Faber M., Ghossein C., Sprague S.M. (2014). A randomized multicenter trial of paricalcitol versus calcitriol for secondary hyperparathyroidism in stages 3-4 CKD. Clin. J. Am. Soc. Nephrol..

[112] Baker L.R., Abrams L., Roe C.J., Faugere M.C., Fanti P., Subayti Y., Malluche H.H. (1989). 1,25(OH)2D3 administration in moderate renal failure: A prospective double-blind trial. Kidney Int..

[113] Mangoo-Karim R., Da Silva Abreu J., Yanev G.P., Perez N.N., Stubbs J.R., Wetmore J.B. (2015). Ergocalciferol versus Cholecalciferol for Nutritional Vitamin D Replacement in CKD. Nephron.

[114] Wetmore J.B., Kimber C., Mahnken J.D., Stubbs J.R. (2016). Cholecalciferol v. ergocalciferol for 25-hydroxyvitamin D (25(OH)D) repletion in chronic kidney disease: A randomised clinical trial. Br. J. Nutr..

[115] Banerjee D., Chitalia N., Ster I.C., Appelbaum E., Thadhani R., Kaski J.C., Goldsmith D. (2019). Impact of Vitamin D on Cardiac structure and function in CKD patients with hypovitaminosis D, a randomised controlled trial and meta-analysis. Eur. Heart J. Cardiovasc. Pharmacother..

[116] Franca Gois P.H., Wolley M., Ranganathan D., Seguro A.C. (2018). Vitamin D Deficiency in Chronic Kidney Disease: Recent Evidence and Controversies. Int. J. Environ. Res. Public Health.

[117] Alshayeb H.M., Josephson M.A., Sprague S.M. (2013). CKD-mineral and bone disorder management in kidney transplant recipients. Am. J. Kidney Dis..

[118] Lu K.C., Ma W.Y., Yu J.C., Wu C.C., Chu P. (2012). Bone turnover markers predict changes in bone mineral density after parathyroidectomy in patients with renal hyperparathyroidism. Clin. Endocrinol. (Oxf).

[119] Nakamura T., Imai Y., Matsumoto T., Sato S., Takeuchi K., Igarashi K., Harada Y., Azuma Y., Krust A., Yamamoto Y. (2007). Estrogen prevents bone loss via estrogen receptor alpha and induction of Fas ligand in osteoclasts. Cell.

[120] Drake M.T., Clarke B.L., Khosla S. (2008). Bisphosphonates: Mechanism of action and role in clinical practice. Mayo Clin. Proc..

[121] Tsoumpra M.K., Muniz J.R., Barnett B.L., Kwaasi A.A., Pilka E.S., Kavanagh K.L., Evdokimov A., Walter R.L., Von Delft F., Ebetino F.H. (2015). The inhibition of human farnesyl pyrophosphate synthase by nitrogen-containing bisphosphonates. Elucidating the role of active site threonine 201 and tyrosine 204 residues using enzyme mutants. Bone.

[122] Miller P.D., Roux C., Boonen S., Barton I.P., Dunlap L.E., Burgio D.E. (2005). Safety and efficacy of risedronate in patients with age-related reduced renal function as estimated by the Cockcroft and Gault method: A pooled analysis of nine clinical trials. J. Bone Miner. Res..

[123] Shigematsu T., Muraoka R., Sugimoto T., Nishizawa Y. (2017). Risedronate therapy in patients with mild-to-moderate chronic kidney disease with osteoporosis: Post-hoc analysis of data from the risedronate phase III clinical trials. BMC Nephrol..

[124] Jamal S.A., Bauer D.C., Ensrud K.E., Cauley J.A., Hochberg M., Ishani A., Cummings S.R. (2007). Alendronate treatment in women with normal to severely impaired renal function: An analysis of the fracture intervention trial. J. Bone Miner. Res..

[125] Toussaint N.D., Lau K.K., Strauss B.J., Polkinghorne K.R., Kerr P.G. (2010). Effect of alendronate on vascular calcification in CKD stages 3 and 4: A pilot randomized controlled trial. Am. J. Kidney Dis..

[126] Black D.M., Delmas P.D., Eastell R., Reid I.R., Boonen S., Cauley J.A., Cosman F., Lakatos P., Leung P.C., Man Z. (2007). Once-yearly zoledronic acid for treatment of postmenopausal osteoporosis. N. Engl. J. Med..

[127] Bergner R., Henrich D., Hoffmann M., Schmidt-Gayk H., Lenz T., Upperkamp M. (2008). Treatment of reduced bone density with ibandronate in dialysis patients. J. Nephrol..

[128] Wilson L.M., Rebholz C.M., Jirru E., Liu M.C., Zhang A., Gayleard J., Chu Y., Robinson K.A. (2017). Benefits and Harms of Osteoporosis Medications in Patients With Chronic Kidney Disease: A Systematic Review and Meta-analysis. Ann. Intern. Med..

[129] Wang J., Yao M., Xu J.H., Shu B., Wang Y.J., Cui X.J. (2016). Bisphosphonates for prevention of osteopenia in kidney-transplant recipients: A systematic review of randomized controlled trials. Osteoporos. Int..

[130] Toth-Manikowski S.M., Francis J.M., Gautam A., Gordon C.E. (2016). Outcomes of bisphosphonate therapy in kidney transplant recipients: A systematic review and meta-analysis. Clin. Transplant..

[131] Lacey D.L., Boyle W.J., Simonet W.S., Kostenuik P.J., Dougall W.C., Sullivan J.K., San Martin J., Dansey R. (2012). Bench to bedside: Elucidation of the OPG-RANK-RANKL pathway and the development of denosumab. Nat. Rev. Drug Discov..

[132] Sobacchi C., Menale C., Villa A. (2019). The RANKL-RANK Axis: A Bone to Thymus Round Trip. Front. Immunol..

[133] Kong Y.Y., Yoshida H., Sarosi I., Tan H.L., Timms E., Capparelli C., Morony S., Oliveira-dos-Santos A.J., Van G., Itie A. (1999). OPGL is a key regulator of osteoclastogenesis, lymphocyte development and lymph-node organogenesis. Nature.

[134] Viswanathan M., Reddy S., Berkman N., Cullen K., Middleton J.C., Nicholson W.K., Kahwati L.C. (2018). U.S. Preventive Services Task Force Evidence Syntheses, formerly Systematic Evidence Reviews. Screening to Prevent Osteoporotic Fractures: An Evidence Review for the US Preventive Services Task Force.

[135] Fontalis A., Kenanidis E., Prousali E., Potoupnis M., Tsiridis E. (2018). Safety and efficacy of denosumab in osteoporotic patients previously treated with other medications: A systematic review and meta-analysis. Expert Opin. Drug Saf..

[136] Mori T., Crandall C.J., Ganz D.A. (2017). Cost-effectiveness of denosumab versus oral alendronate for elderly osteoporotic women in Japan. Osteoporos. Int..

[137] Beaudoin C., Jean S., Bessette L., Ste-Marie L.G., Moore L., Brown J.P. (2016). Denosumab compared to other treatments to prevent or treat osteoporosis in individuals at risk of fracture: A systematic review and meta-analysis. Osteoporos. Int..

[138] Samelson E.J., Miller P.D., Christiansen C., Daizadeh N.S., Grazette L., Anthony M.S., Egbuna O., Wang A., Siddhanti S.R., Cheung A.M. (2014). RANKL inhibition with denosumab does not influence 3-year progression of aortic calcification or incidence of adverse cardiovascular events in postmenopausal women with osteoporosis and high cardiovascular risk. J. Bone Miner. Res..

[139] Block G.A., Bone H.G., Fang L., Lee E., Padhi D. (2012). A single-dose study of denosumab in patients with various degrees of renal impairment. J. Bone Miner. Res..

[140] Jamal S.A., Ljunggren O., Stehman-Breen C., Cummings S.R., McClung M.R., Goemaere S., Ebeling P.R., Franek E., Yang Y.C., Egbuna O.I. (2011). Effects of denosumab on fracture and bone mineral density by level of kidney function. J. Bone Miner. Res..

[141] Fraser T.R., Flogaitis I., Moore A.E., Hampson G. (2020). The effect of previous treatment with bisphosphonate and renal impairment on the response to denosumab in osteoporosis: A ‘real-life’ study. J. Endocrinol. Investig..

[142] Chen C.L., Chen N.C., Hsu C.Y., Chou K.J., Lee P.T., Fang H.C., Renn J.H. (2014). An open-label, prospective pilot clinical study of denosumab for severe hyperparathyroidism in patients with low bone mass undergoing dialysis. J. Clin. Endocrinol. Metab..

[143] Festuccia F., Jafari M.T., Moioli A., Fofi C., Barberi S., Amendola S., Sciacchitano S., Punzo G., Mene P. (2017). Safety and efficacy of denosumab in osteoporotic hemodialysed patients. J. Nephrol..

[144] Salim S.A., Nair L.R., Thomas L., Garla V., Palabindala V., Agarwal M., Fulop T. (2018). Denosumab-Associated Severe Hypocalcemia in a Patient with Chronic Kidney Disease. Am. J. Med. Sci..

[145] McCormick B.B., Davis J., Burns K.D. (2012). Severe hypocalcemia following denosumab injection in a hemodialysis patient. Am. J. Kidney Dis..

[146] Shrosbree J.E., Elder G.J., Eisman J.A., Center J.R. (2018). Acute hypocalcaemia following denosumab in heart and lung transplant patients with osteoporosis. Intern. Med. J..

[147] Nanmoku K., Shinzato T., Kubo T., Shimizu T., Yagisawa T. (2019). Effects of denosumab on hypercalcemia and bone mineral density loss in kidney transplant recipients. Clin. Nephrol..

[148] Thongprayoon C., Acharya P., Aeddula N.R., Torres-Ortiz A., Bathini T., Sharma K., Ungprasert P., Watthanasuntorn K., Suarez M.L.G., Salim S.A. (2019). Effects of denosumab on bone metabolism and bone mineral density in kidney transplant patients: A systematic review and meta-analysis. Arch. Osteoporos..

[149] McKee H., Ioannidis G., Lau A., Treleaven D., Gangji A., Ribic C., Wong-Pack M., Papaioannou A., Adachi J.D. (2020). Correction to: Comparison of the clinical effectiveness and safety between the use of denosumab vs bisphosphonates in renal transplant patients. Osteoporos. Int..

[150] Kobel C., Frey D., Graf N., Wuthrich R.P., Bonani M. (2019). Follow-Up of Bone Mineral Density Changes in de novo Kidney Transplant Recipients Treated with Two Doses of the Receptor Activator of Nuclear Factor kappaB Ligand Inhibitor Denosumab. Kidney Blood Press. Res..

[151] Dave V., Chiang C.Y., Booth J., Mount P.F. (2015). Hypocalcemia post denosumab in patients with chronic kidney disease stage 4–5. Am. J. Nephrol..

[152] Nitta K., Yajima A., Tsuchiya K. (2017). Management of Osteoporosis in Chronic Kidney Disease. Intern. Med..

[153] Bhanot R.D., Kaur J., Bhat Z. (2019). Severe Hypocalcemia and Dramatic Increase in Parathyroid Hormone after Denosumab in a Dialysis Patient: A Case Report and Review of the Literature. Case Rep. Nephrol..

[154] Huynh A.L., Baker S.T., Stewardson A.J., Johnson D.F. (2016). Denosumab-associated hypocalcaemia: Incidence, severity and patient characteristics in a tertiary hospital setting. Pharm. Drug Saf..

[155] Hamano T., Nakano C. (2016). Is denosmab really effective and safe in the care of CKD-MBD?. Clin. Calcium.

[156] Black D.M., Rosen C.J. (2016). Clinical Practice. Postmenopausal Osteoporosis. N. Engl. J. Med..

[157] McDonnell D.P. (2003). Mining the complexities of the estrogen signaling pathways for novel therapeutics. Endocrinology.

[158] Ishani A., Blackwell T., Jamal S.A., Cummings S.R., Ensrud K.E., Investigators M. (2008). The effect of raloxifene treatment in postmenopausal women with CKD. J. Am. Soc. Nephrol..

[159] Haghverdi F., Farbodara T., Mortaji S., Soltani P., Saidi N. (2014). Effect of raloxifene on parathyroid hormone in osteopenic and osteoporotic postmenopausal women with chronic kidney disease stage 5. Iran. J. Kidney Dis..

[160] Hernandez E., Valera R., Alonzo E., Bajares-Lilue M., Carlini R., Capriles F., Martinis R., Bellorin-Font E., Weisinger J.R. (2003). Effects of raloxifene on bone metabolism and serum lipids in postmenopausal women on chronic hemodialysis. Kidney Int..

[161] Zanchetta J.R., Bogado C.E., Ferretti J.L., Wang O., Wilson M.G., Sato M., Gaich G.A., Dalsky G.P., Myers S.L. (2003). Effects of teriparatide [recombinant human parathyroid hormone (1-34)] on cortical bone in postmenopausal women with osteoporosis. J. Bone Miner. Res..

[162] Gardella T.J., Juppner H. (2001). Molecular properties of the PTH/PTHrP receptor. Trends Endocrinol. Metab..

[163] Hodsman A.B., Bauer D.C., Dempster D.W., Dian L., Hanley D.A., Harris S.T., Kendler D.L., McClung M.R., Miller P.D., Olszynski W.P. (2005). Parathyroid hormone and teriparatide for the treatment of osteoporosis: A review of the evidence and suggested guidelines for its use. Endocr. Rev..

[164] Cejka D., Jager-Lansky A., Kieweg H., Weber M., Bieglmayer C., Haider D.G., Diarra D., Patsch J.M., Kainberger F., Bohle B. (2012). Sclerostin serum levels correlate positively with bone mineral density and microarchitecture in haemodialysis patients. Nephrol. Dial. Transplant..

[165] Cejka D., Herberth J., Branscum A.J., Fardo D.W., Monier-Faugere M.C., Diarra D., Haas M., Malluche H.H. (2011). Sclerostin and Dickkopf-1 in renal osteodystrophy. Clin. J. Am. Soc. Nephrol..

[166] Black D.M., Bilezikian J.P., Ensrud K.E., Greenspan S.L., Palermo L., Hue T., Lang T.F., McGowan J.A., Rosen C.J., PaTH Study Investigators (2005). One year of alendronate after one year of parathyroid hormone (1-84) for osteoporosis. N. Engl. J. Med..

[167] Miller P.D., Schwartz E.N., Chen P., Misurski D.A., Krege J.H. (2007). Teriparatide in postmenopausal women with osteoporosis and mild or moderate renal impairment. Osteoporos. Int..

[168] Nishikawa A., Ishida T., Taketsuna M., Yoshiki F., Enomoto H. (2016). Safety and effectiveness of daily teriparatide in a prospective observational study in patients with osteoporosis at high risk of fracture in Japan: Final report. Clin. Interv. Aging.

[169] Cejka D., Kodras K., Bader T., Haas M. (2010). Treatment of Hemodialysis-Associated Adynamic Bone Disease with Teriparatide (PTH1-34): A Pilot Study. Kidney Blood Press. Res..

[170] Palcu P., Dion N., Ste-Marie L.G., Goltzman D., Radziunas I., Miller P.D., Jamal S.A. (2015). Teriparatide and bone turnover and formation in a hemodialysis patient with low-turnover bone disease: A case report. Am. J. Kidney Dis..

[171] Giamalis P., Economidou D., Dimitriadis C., Memmos D., Papagianni A., Efstratiadis G. (2015). Treatment of adynamic bone disease in a haemodialysis patient with teriparatide. Clin. Kidney J..

[172] Sumida K., Ubara Y., Hoshino J., Mise K., Hayami N., Suwabe T., Kawada M., Imafuku A., Hiramatsu R., Hasegawa E. (2016). Once-weekly teriparatide in hemodialysis patients with hypoparathyroidism and low bone mass: A prospective study. Osteoporos. Int..

[173] Cejka D., Benesch T., Krestan C., Roschger P., Klaushofer K., Pietschmann P., Haas M. (2008). Effect of teriparatide on early bone loss after kidney transplantation. Am. J. Transplant..

[174] Hattersley G., Dean T., Corbin B.A., Bahar H., Gardella T.J. (2016). Binding Selectivity of Abaloparatide for PTH-Type-1-Receptor Conformations and Effects on Downstream Signaling. Endocrinology.

[175] Miller P.D., Hattersley G., Riis B.J., Williams G.C., Lau E., Russo L.A., Alexandersen P., Zerbini C.A., Hu M.Y., Harris A.G. (2016). Effect of Abaloparatide vs. Placebo on New Vertebral Fractures in Postmenopausal Women With Osteoporosis: A Randomized Clinical Trial. JAMA.

[176] Leder B.Z., O’Dea L.S., Zanchetta J.R., Kumar P., Banks K., McKay K., Lyttle C.R., Hattersley G. (2015). Effects of abaloparatide, a human parathyroid hormone-related peptide analog, on bone mineral density in postmenopausal women with osteoporosis. J. Clin. Endocrinol. Metab..

[177] Pietrzyk B., Smertka M., Chudek J. (2017). Sclerostin: Intracellular mechanisms of action and its role in the pathogenesis of skeletal and vascular disorders. Adv. Clin. Exp. Med..

[178] McClung M.R., Grauer A., Boonen S., Bolognese M.A., Brown J.P., Diez-Perez A., Langdahl B.L., Reginster J.Y., Zanchetta J.R., Wasserman S.M. (2014). Romosozumab in postmenopausal women with low bone mineral density. N. Engl. J. Med..

[179] Bandeira L., Lewiecki E.M., Bilezikian J.P. (2017). Romosozumab for the treatment of osteoporosis. Expert Opin. Biol. Ther..

[180] Cosman F., Crittenden D.B., Adachi J.D., Binkley N., Czerwinski E., Ferrari S., Hofbauer L.C., Lau E., Lewiecki E.M., Miyauchi A. (2016). Romosozumab Treatment in Postmenopausal Women with Osteoporosis. N. Engl. J. Med..

[181] Brandenburg V.M., Verhulst A., Babler A., D’Haese P.C., Evenepoel P., Kaesler N. (2019). Sclerostin in chronic kidney disease-mineral bone disorder think first before you block it!. Nephrol. Dial. Transplant..

